# Silibinin Suppresses Inflammatory Responses Induced by Exposure to Asian Sand Dust

**DOI:** 10.3390/antiox13101187

**Published:** 2024-09-30

**Authors:** Se-Jin Lee, So-Won Pak, Woong-Il Kim, Sin-Hyang Park, Young-Kwon Cho, Je-Won Ko, Tae-Won Kim, Joong-Sun Kim, Jong-Choon Kim, Je-Oh Lim, In-Sik Shin

**Affiliations:** 1 BK21 FOUR Program, College of Veterinary Medicine, Chonnam National University, 77 Yong-bong-ro, Buk-gu, Gwangju 61186, Republic of Korea; 218729@jnu.ac.kr (S.-J.L.); dvmpsw@jnu.ac.kr (S.-W.P.); dvmwoong@jnu.ac.kr (W.-I.K.); sinhyang23@jnu.ac.kr (S.-H.P.); centraline@jnu.ac.kr (J.-S.K.); toxkim@jnu.ac.kr (J.-C.K.); 2 College of Health Sciences, Cheongju University, 298 Daesung-ro, Sangdang-gu, Cheongju-si 28503, Republic of Korea; petmen@cju.ac.kr; 3 BK21 FOUR Program, College of Veterinary Medicine, Chungnam National University, 99 Daehak-ro, Daejeon 34134, Republic of Korea; rheoda@cnu.ac.kr (J.-W.K.); taewonkim@cnu.ac.kr (T.-W.K.); 4 Herbal Medicine Resources Research Center, Korea Institute of Oriental Medicine, 177 Geonjae-ro, Naju-si 58245, Republic of Korea

**Keywords:** silibinin, Asian sand dust, pulmonary inflammation, p-p65, Heme oxygenase-1

## Abstract

Asian sand dust (ASD), generated from the deserts of China and Mongolia, affects Korea and Japan during spring and autumn, causing harmful effects on various bio-organs, including the respiratory system, due to its irritants such as fine dust, chemicals, and toxic materials. Here, we investigated the therapeutic effects of silibinin against ASD-induced airway inflammation using mouse macrophage-like cell line RAW264.7 and a murine model. ASD was intranasally administered to mice three times a week and silibinin was administered for 6 days by oral gavage. In ASD-stimulated RAW264.7 cells, silibinin treatment decreased tumor necrosis factor-α production and reduced the expression of p-p65NF-κB, p-p38, and cyclooxygenase (COX)-2, while increasing heme oxygenase (HO)-1 expression. In ASD-exposed mice, silibinin administration reduced inflammatory cell count and cytokines in bronchoalveolar lavage fluid and decreased inflammatory cell infiltration in lung tissue. Additionally, silibinin lowered oxidative stress, as evidenced by decreased 8-hydroxy-2’-deoxyguanosin (8-OHdG) expression and increased HO-1 expression. The expression of inflammatory-related proteins, including p-p65NF-κB, COX-2, and p-p38, was markedly reduced by silibinin administration. Overall, silibinin treatment reduced the expression of p-p65NF-κB, COX-2, and p-p38 in response to ASD exposure, while increasing HO-1 expression both in vitro and in vivo. These findings suggest that silibinin mitigates pulmonary inflammation caused by ASD exposure by reducing inflammatory signaling and oxidative stress, indicating its potential as a therapeutic agent for ASD-induced pulmonary inflammation.

## 1. Introduction

Asian sand dust (ASD), mainly generated from the deserts of Mongolia and China, affects East Asia, including China, Japan, and the Republic of Korea [1,2]. In particular, ASD occurs in spring and autumn, periods when respiratory diseases develop frequently due to decreased immunity caused by temperature changes, further increasing the prevalence of respiratory diseases and worsening underlying respiratory diseases such as allergic asthma and chronic obstructive pulmonary disease [3,4]. Due to rapid industrialization, ASD contains heavy metals and nanomaterials harmful to organisms, threatening human health [5,6]. Therefore, ASD is a very important issue for human respiratory health in these regions. In the Republic of Korea, information on air quality is released to the public every day, and recommendations on wearing masks and refraining from going out are implemented.

Silibinin is the main component of *Silybum marianum* (milk thistle), which is mainly distributed throughout Europe, Russia, and Central Asia [7]. Currently, milk thistle is used as a functional food against various diseases due to its pharmacological properties [8]. Furthermore, the anticancer, anti-inflammatory, antioxidative, and antifibrotic effects of silibinin have been proven by many experimental studies [9,10,11]. Thus, silibinin has been used to treat various diseases, including cancer, diabetes, Alzheimer’s disease, and hepatic diseases [7]. Additionally, silibinin is used to treat many inflammatory diseases, including dermatitis, sepsis, hepatitis, and arthritis [12,13,14,15]. Recently, silibinin has demonstrated therapeutic properties for various pulmonary diseases, including chronic obstructive pulmonary disease, asthma, lung fibrosis, and airway inflammation induced by metals and nanoparticles [16,17,18,19,20]. Consequently, we hypothesized that silibinin would be effective against ASD-induced pulmonary inflammation.

While previous studies have suggested the respiratory toxicity of ASD, studies focusing on the use of protective agents against ASD-induced airway inflammation remain limited. This study investigated the therapeutic effects of silibinin on ASD-induced pulmonary inflammation using in vivo and in vitro experimental models. Additionally, we elucidated the underlying mechanism of silibinin by analyzing the expression of proteins related to oxidative stress and inflammatory responses.

## 2. Materials and Methods

### 2.1. Physical and Chemical Characteristics of ASD

The morphology and composition of Asian sand dust (ASD; Powder Technology Inc., Arden Hills, MN, USA) were measured using transmission electron microscopy (TEM; JEM-2100 F, JEOL, Tokyo, Japan) and scanning electron microscopy (SEM; Zeiss Gemini500, Carl Zeiss Meditec AG, Jena, Germany), respectively. Its chemical elements consist of 34.0–40.0% SiO_2_, 26.0–32.0% Al_2_O_3_, 17.0–23.0% Fe_2_O_3_, 3.0–7.0% MgO, 0.0–3.0% CaO, and 0.0–4.0% TiO_2_. The ASD’s purity was evaluated using energy dispersive X-ray spectroscopy (X-Max N 150 mm^2^ silicon drift detector equipped with a Zeiss Gemini500 SEM; Oxford Instrument, Abingdon, UK). The hydrodynamic size of ASD was evaluated using ELS-8000 (Otsuka Electronic, Tokyo, Japan).

### 2.2. Procedure for Animal Experiment

C57BL/6 male mice (6 weeks old, Samtako Co., Osan, Republic of Korea) were housed at a temperature of 22 ± 2 °C, humidity of 55 ± 5%, and a photoperiod of 12 h night/day, and feed and water were provided *ad libitum*. The animals were divided into the following 4 groups (*n* = 7 per group): a normal control group (NC group), an ASD-induced pulmonary inflammation group (ASD group), and silibinin-treated groups (Sil 20 group and Sil 40 group). The murine model of pulmonary inflammation induced by ASD exposure was established according to a previous study’s methodology [2]. The instillation dose of ASD (0.8 mg/mouse) used in this study was 13.3 times higher than the approximately 60 µg associated with 100% deposition, according to the Korean national air quality standard for suspended particulate matter (PM) [21]. However, during high-ASD-level days in the Republic of Korea, the national air quality standard for suspended PM generally exceeds 150 µg/m^3^ [22]. Therefore, the instillation dose of ASD was 5.3 times higher. ASD (0.8 mg/mouse in 50 µL of phosphate buffered saline ((PBS)) was administered to animals in the ASD and Sil groups via intranasal instillation on days 1, 3, and 5 under slight anesthesia. The NC group was intranasally instilled with 50 µL of PBS. Silibinin (20 and 40 mg/kg in PBS) was administered to animals in the Sil groups daily from days 1 to 6 by oral gavage. The animals were sacrificed 48 h after their last ASD exposure.

To collect the bronchoalveolar lavage fluid (BALF) from animals, we performed tracheostomy on each animal, according to the methodology of a previous study [23]. First, the trachea of the animal was incised and then an endotracheal tube was inserted. The lung was lavaged twice with 0.7 mL of phosphate-buffered saline (PBS, total volume: 1.4 mL), which was then centrifuged at 1500 rpm for 5 min at 4 °C. The supernatant was collected and stored at −80 °C to analyze inflammatory cytokines, including interleukin (IL)-1β, IL-6 and tumor necrosis factor (TNF)-α, using enzyme-linked immunosorbent assay (ELISA) kits (BD Biosciences, San Jose, CA, USA) according to the manufacturer’s protocols. The amount of inflammatory cytokines was calculated according to the measured absorbance using the standard curve. The absorbance was measured at 450 nm using a spectrophotometer (Thermo Fisher Scientific, San Diego, CA, USA). The pellet was used to determine the number of inflammatory cells in the BALF. The pellet was resuspended in PBS and the number of total inflammatory cells in the BALF was determined using an automated cell counter (Countess II, Thermo Fisher Scientific, San Diego, CA, USA). To count the number of differential inflammatory cells in the BALF, a resuspended pellet (200 μL) was attached onto a glass slide using a cytospin (Hanil Scientific Inc., Gimpo, Republic of Korea) and then stained with Diff-Quik agent (Sysmex, Kobe, Japan). The number of inflammatory cells, including neutrophils, macrophages, eosinophils and lymphocytes, were counted under a light microscope (Leica Microsystem, Heidelberg, Germany), and the ratio of differential inflammatory cells out of the total inflammatory cells was determined.

The procedure for the animal experiment was checked by the Chonnam National University Institutional Animal Care and Use Committee (CNU IACUC-YB-2022-123). The animals were cared for in accordance with the Guideline for Animal Experiments of Chonnam National University.

### 2.3. Histological Examination

The left lung, after BALF sampling, was fixed and neutralized in 4% formalin for 3 days at room temperature. The tissues were embedded in a paraffin block, cut into 4 μm thick sections, deparaffinized using xylene, and dehydrated using ethanol. Following a 5 min wash with distilled water, tissue sections were stained with hematoxylin and eosin stain (H&E; Sigma-Aldrich, St. Louis, MO, USA). To investigate the protein expression related to oxidative stress, we performed immunohistochemistry (IHC) analysis according to a previous study’s methodology [24]. IHC was performed using a commercial kit (Vector Laboratories, Burlingame, CA, USA) and the following primary antibodies were used: anti-rabbit heme oxygenase (HO)-1 (Abcam, Cambridge, UK) and anti-mouse 8-OHdG (Santa Cruz Biotechnology, Santa Cruz, CA, USA). To perform immunohistochemistry (IHC) analysis, deparaffinized and dehydrated tissues were washed with PBS-T (PBS containing 0.05% Tween 20) and incubated for 20 min with goat serum at room temperature to block nonspecific protein binding. The tissues were incubated with primary antibodies for 2 h at room temperature, washed 3 times, and incubated with biotinylated secondary antibody for 1 h at room temperature. Tissues were then incubated with an avidin–biotin–peroxidase complex (Vector Laboratories, Burlingame, CA, USA) for 1 h at room temperature and further incubated with diaminobenzidine (DAB, Abcam, Cambridge, UK) for 5 min before visualizing using a light microscope (Leica Microsystem). The representative figures for histological examination were obtained using a slide scanner (Motic, Richmond, BC, Canada) and the quantification of respiratory inflammatory responses and protein expression were carried out using an Image and Microscope Technology (IMT) i-Solution, as an image analyzer (IMT i-Solution Inc., Vancouver, BC, Canada). For the quantitative analysis of inflammation, we evaluated the inflammatory response for the total area (captured on 200× magnification). The quantitative value was expressed as a percentage (%, inflammation or mucus secretion area vs. arranged area). In addition, we performed an immunofluorescence analysis to evaluate p-p65 expression using anti-mouse p-p65 (1:200; Cell Signaling, Danver, MA, USA) according to a previous study’s methodology [25]. The slides were mounted using Prolong Gold antifade with 4’,6-diamidino-2-phenylindole (DAPI, Thermo Fisher Scientific, San Diego, CA, USA) and evaluated using confocal microscopy (Carl Zeiss, Oberkochen, Germany).

### 2.4. Western Blot Analysis

The right lung tissue was homogenized (1/10 *w*/*v*) in tissue lysis/extraction reagent (Sigma-Aldrich, St. Louis, MO, USA) supplemented with protease inhibitors (Sigma-Aldrich, St. Louis, MO, USA) to determine protein expression and pathways. Western blotting was performed as described previously [25]. Each sample (30 μg/well) was separated using 10% sodium dodecyl sulfate-polyacrylamide gel electrophoresis (SDS-PAGE) and transferred to 0.25 μm polyvinylidene difluoride (PVDF) membranes (Millipore, Burlington, MA, USA). The membranes were then blocked with 5% skimmed milk at room temperature for 2 h, before being incubated with primary antibodies overnight. The following primary antibodies were used: phospho-p38 (1:1000; Cell Signaling, Danvers, MA, USA), p-p65 (1:1000; Cell Signaling, Danvers, MA, USA), cyclooxygenase (COX)-2 (1:1000; Abcam, Cambridge, UK), HO-1 (1:1000 dilution, Abcam, Cambridge, UK), and β-actin (1:1000; Cell Signaling, Danvers, MA, USA). Then, the membranes were incubated with a horseradish peroxidase (HRP)-conjugated secondary antibody (1:10,000; Jackson Immuno Research, West Grove, PA, USA) for 2 h, and finally developed using an enhanced chemiluminescence kit (Thermo Fisher Scientific, San Diego, CA, USA). The densitometric values of the protein band expressions were assessed using ChemiDoc (Bio-Rad Laboratories, Hercules, CA, USA).

### 2.5. Cell Culture

RAW264.7 cells were cultured using Dulbecco’s modified Eagle’s medium (Sigma-Aldrich, St. Louis, MO, USA) supplemented with 10% fetal bovine serum (FBS, Sigma-Aldrich, St. Louis, MO, USA) and 1% antibiotics (Sigma-Aldrich, St. Louis, MO, USA) at 37 °C in a 5% CO_2_ incubator (Thermo Fisher Scientific, San Diego, CA, USA). Cell viability was evaluated using EZ-Cytox (DAELIL lab, Seoul, Republic of Korea). The cells (1 × 10^4^ cells/well) were seeded in 96-well plates and incubated for 24 h. Fresh medium and various concentrations of silibinin (20, 10, 5, 2.5, 1.25, and 0.725 µg/mL) were added after 24 h. Each well was treated with EZ-Cytox agent and incubated for 4 h, and then absorbance was measured at 450 nm using a spectrophotometer (Thermo Fisher Scientific, San Diego, CA, USA).

### 2.6. Measurement of TNF-α in ASD-Stimulated RAW264.7 Cells

The cells (1 × 10^4^ cells/well) were seeded in 6-well plates and treated with various concentrations of silibinin. After 1 h, ASD (20 µg/mL) was added to each well and incubated for 24 h. The supernatants of cells were collected to measure the production of TNF-α (R&D Systems, Minneapolis, MN, USA) according to the manufacturer’s procedure. The absorbance was measured at 450 nm using a spectrophotometer (Thermo Fisher Scientific, San Diego, CA, USA).

### 2.7. Measurement of p-p65 Expression in ASD-Stimulated RAW264.7 Cells

Double-immunofluorescence (IF) analysis was conducted to determine p-p65 expression, as previously described [25], using an anti-p-p65 (1:100; Abcam, Cambridge, UK) antibody. RAW264.7 cells were seeded in 12-well plates with plastic coverslips (SPL Life Sciences, Pocheon, Republic of Korea). The cells attached to the plastic coverslip were exposed to ASD, and the cells cultured for 6 h were used for double IF. The cells were incubated with the primary antibody overnight at 4 °C and the secondary antibody for 2 h at room temperature. After 3 washes in PBS, the cells were incubated with anti-rabbit fluorescein isothiocyanate (FITC) secondary antibody (Sigma-Aldrich, St. Louis, MO, USA). The cells were mounted on slides and the nuclei were visualized with 4’,6-diamidino-2-phenylindole (DAPI) present in the ProLong Gold Antifade Mounting Medium (Invitrogen, Carlsbad, CA, USA). Immunofluorescence imaging was performed on a laser scanning confocal microscope (Carl Zeiss, Oberkochen, Germany) using a Leica 63× (N.A. 1.4) oil objective lens.

### 2.8. Statistical Analysis

The results are expressed as mean ± standard deviation. Statistical significance was determined using Dunnett’s test after a one-way ANOVA was conducted using GraphPad Prism 8 (GraphPad Software, San Diego, CA, USA). A *p* value of <0.05 indicated statistical significance (^#^ vs. NC and * vs. ASD).

## 3. Results

### 3.1. Characteristics of ASD

**Figure 1:**
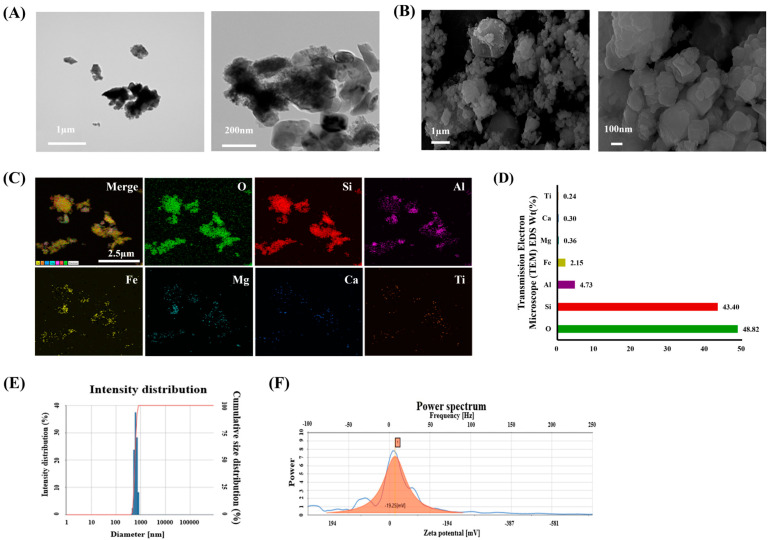
Characteristics of Asian sand dust (ASD). (**A**) Representative figure of scanning electron microscopy (SEM) for ASD, (**B**) representative figure of transmission electron microscopy (TEM) for ASD, (**C**) representative figure of ASD composition analyzed using X-ray spectroscopy on SEM images, (**D**) quantitative analysis of ASD composition, (**E**) average particle size of ASD in distilled water, and (**F**) surface charge of ASD as measured using zeta potential.

The shape and size of ASD particles were analyzed using SEM and TEM (Figure 1A,B, respectively). The constituent elements of ASD were 48.82% O, 43.40% Si, 4.73% Al, 2.15% Fe, 0.36% Mg, 0.30% Ca, and 0.24% Ti (Figure 1C,D). The autocorrelation function and intensity distribution of ASD were measured using the zeta potential (Figure 1E). The average negative zeta potential was −19.25 (mV) (Figure 1F).

### 3.2. Silibinin Decreased Inflammatory Responses in ASD-Stimulated RAW264.7 Cells

**Figure 2:**
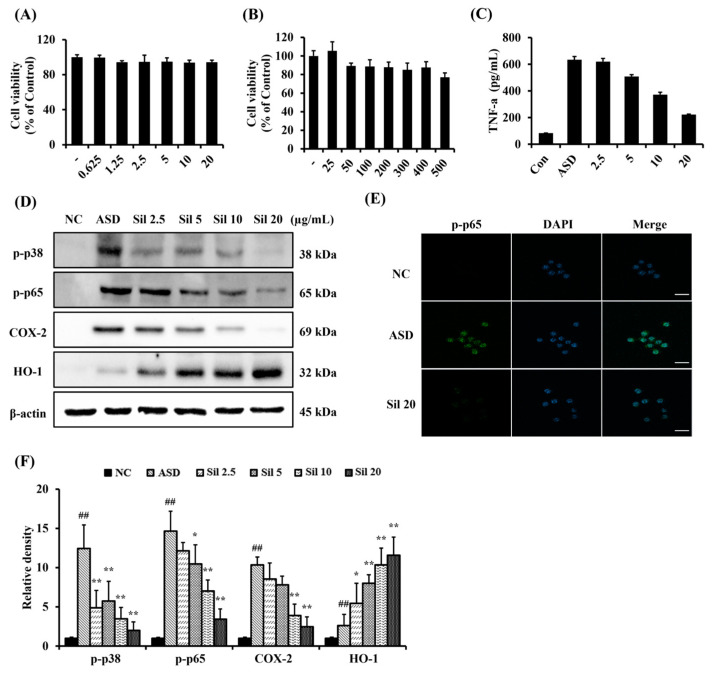
Effects of silibinin on inflammatory responses in ASD-stimulated RAW264.7 cells. (**A**) silibinin cell viability, (**B**) ASD cell viability, (**C**) level of TNF-α, (**D**) Western blot analysis Re-sults, (**E**) representative figure showing immunofluorescence for p-p65, (scale bar: 50 μm), and (**F**) densitometric value of protein expression. The data are shown as the mean ± standard deviation. ^##^, vs. NC, *,**, vs. ASD-stimulated RAW264.7 cell, *p* < 0.05 and <0.01, respectively.

Based on the results of the silibinin and ASD cell viability test (Figure 2A,B, respectively), we determined 20 and 500 μg/mL to be the highest concentrations. ASD-stimulated RAW264.7 cells treated with a concentration of 500 μg/mL showed a significantly increased production of TNF-α compared to non-stimulated RAW264.7 cells. By contrast, silibinin treatment significantly decreased TNF-α production caused by ASD exposure in a concentration-dependent manner (Figure 2C). ASD-stimulated RAW264.7 cells had a significantly elevated expression of p-p38, p-p65, and COX-2 compared to non-stimulated RAW264.7 cells. However, silibinin treatment markedly decreased the expression of p-p38, p-p65, and COX-2 (Figure 2D,F, respectively). HO-1 expression was markedly increased in ASD-stimulated RAW264.7 cells compared with non-stimulated RAW264.7 cells, which was increased even further by silibinin treatment. Additionally, ASD-stimulated RAW264.7 cells showed the increased translocation of p65 into the nucleus, compared with non-stimulated RAW264.7 cells. However, silibinin treatment decreased this translocation induced by ASD stimulation (Figure 2E).

### 3.3. Silibinin Reduced Inflammatory Mediators of BALF of ASD-Exposed Mice

**Figure 3:**
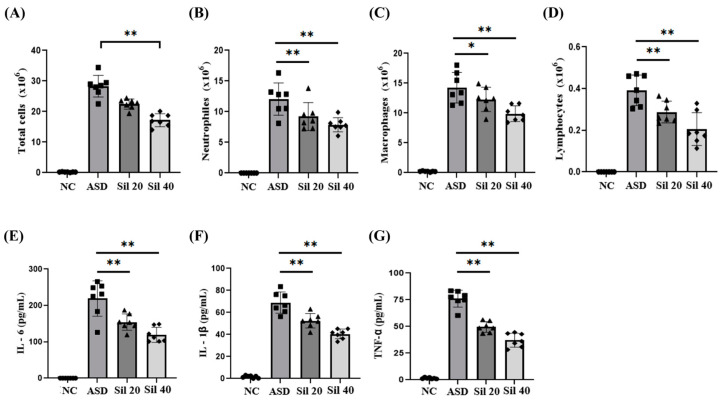
Effects of silibinin on inflammatory cell counts and cytokines in the BALF of ASD-exposed mice. (**A**) Total cells, (**B**) neutrophils, (**C**) macrophages, (**D**) lymphocytes, (**E**) level of IL-6, (**F**) level of IL-1β, and (**G**) level of TNF-α. NC, PBS intranasal instillation + PBS; ASD, ASD intranasal instillation + PBS; Sil 20 and 40, ASD intranasal instillation + silibinin (20 and 40 mg/kg, respectively). The data are presented as the mean ± standard deviation (n = 7). *p* < 0.01; *, **, vs. ASD, *p* < 0.05 and <0.01, respectively.

ASD-exposed mice showed markedly increased total numbers of inflammatory cells in the BALF compared with controls (Figure 3A). Furthermore, the number of neutrophils, macrophages, and lymphocytes were markedly elevated in ASD-exposed mice compared to controls (Figure 3B–D, respectively). In contrast, the silibinin-treated groups demonstrated a significantly decreased number of inflammatory cells in the BALF, in a dose-dependent manner, compared with those of ASD group. The levels of IL-6, IL-1β, and TNF-α in the BALF were markedly increased in ASD-exposed mice compared with the controls (Figure 3E–G, respectively). However, silibinin-treated groups showed significantly decreased levels of IL-6, IL-1β, and TNF-α in the BALF, in a dose-dependent manner, compared with those of ASD-exposed mice.

### 3.4. Silibinin Decreased Inflammatory Response and Oxidative Stress in the Lung Tissue of ASD-Exposed Mice

**Figure 4:**
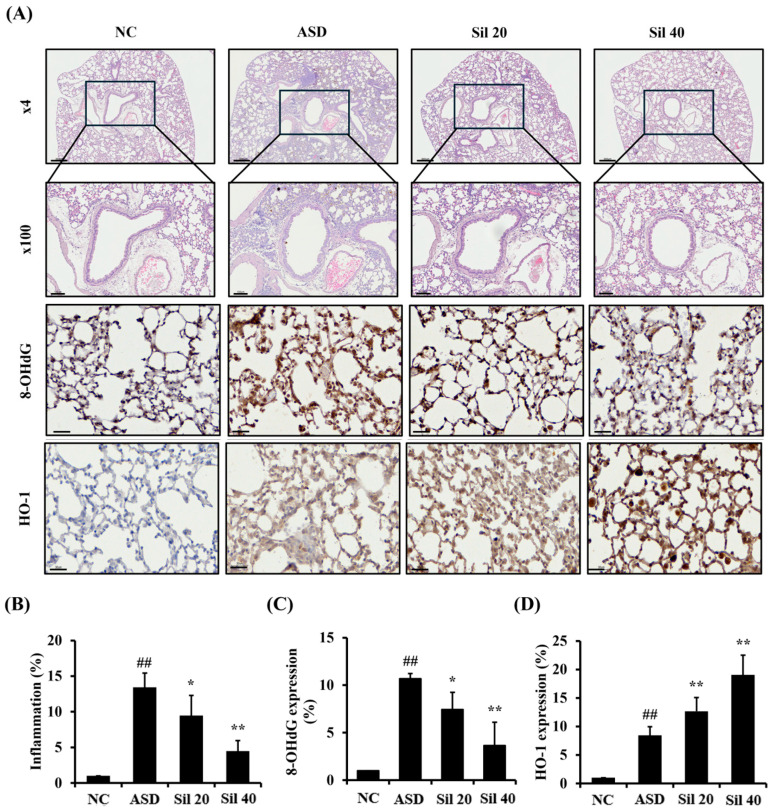
Effects of silibinin on inflammatory responses and oxidative stress in ASD-exposed mice. (**A**) Representative figures for H&E stain and IHC analysis (8-OHdG and HO-1), (**B**) inflammation index, (**C**) 8-OHdG expression value, and (**D**) HO-1 expression value. NC, PBS intranasal instillation + PBS; ASD, ASD intranasal instillation + PBS; Sil 20 and 40, ASD intranasal instillation + silibinin (20 and 40 mg/kg, respectively). The data are presented as the mean ± standard deviation (n = 7). Lung tissue of a mouse captured at 4× magnification (scale bar: 300 µm) and 100× magnification (scale bar: 100 µm). ^##^, vs. NC, *p* < 0.01; *, **, vs. ASD, *p* < 0.05 and <0.01, respectively.

ASD-exposed mice showed extensive inflammatory cell infiltration in the lung tissue, which was significantly decreased by silibinin (Figure 4A,B). The expression of 8-OHdG and HO-1 in the lung tissue markedly increased in ASD-exposed mice compared to controls (Figure 4A,C,D). However, the silibinin-treated group showed a significantly decreased expression of 8-OHdG and an increased expression of HO-1 compared with those of ASD-exposed mice.

### 3.5. Silibinin Suppressed Inflammatory Signaling in ASD-Exposed Mice

**Figure 5:**
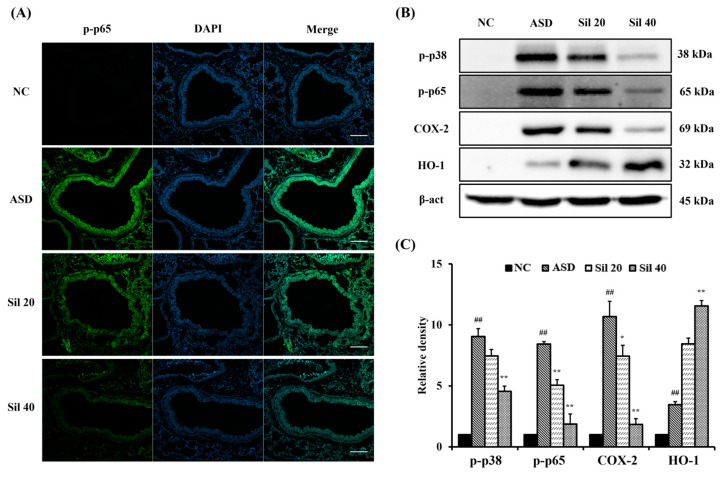
Effects of silibinin on the signaling pathway related to inflammation and oxidative stress in ASD-exposed mice. (**A**) Representative figure of p-p65 expression in the lung tissue (scale bar: 100 μm), (**B**) Western blot analysis results, and (**C**) densitometric value of protein expression. NC, PBS intranasal instillation + PBS; ASD, ASD intranasal instillation + PBS; Sil 20 and 40, ASD intranasal instillation + silibinin (20 and 40 mg/kg, respectively). The data are presented as the mean ± standard deviation (n = 7). ^##^, vs. NC, *p* < 0.01; *, **, vs. ASD, *p* < 0.05 and <0.01, respectively.

ASD-exposed mice showed markedly increased p-p65 expression compared to controls (Figure 5A–C). However, silibinin-treated mice significantly decreased the expression of p-p65 compared with that of ASD-exposed mice. Furthermore, the expression of p-p38, COX-2, and HO-1 was markedly increased in ASD-exposed mice compared to controls (Figure 5B,C, respectively). Silibinin treatment also significantly decreased the expression of p-p38 and COX-2 and increased the expression of HO-1.

## 4. Discussion

ASD affects China, Japan, and the Republic of Korea, threatening people’s respiratory health and worsening underlying diseases [26,27]. Currently, ASD is regarded as an important social problem, and each country is responding by establishing policies to solve various problems caused by ASD exposure [28]. However, the use of therapeutic agents to treat the toxic effects of ASD on the respiratory tract is limited. In this study, we investigated the therapeutic effects of silibinin on pulmonary inflammation induced by ASD exposure. The silibinin treatment resulted in a significant reduction in inflammatory cell counts in the BALF, including neutrophils, macrophages, and lymphocytes, which was accompanied by the decreases in the production of IL-1β, IL-6, and TNF-α. In histological analysis, silibinin treatment resulted in a reduction in the inflammatory responses of the respiratory tract induced by ASD exposure. In addition, silibinin treatment demonstrated antioxidant properties, including the elevation of HO-1 expression and a reduction in 8-OHdG, as well as anti-inflammatory properties, including reductions in the expression of p-p38, p-p65, and COX-2 in lung tissue. These events were consistent with the results of in vitro experiments. Silibinin treatment resulted in a significant reduction in the production of TNF-α in ASD-stimulated RAW264.7 cells, as well as an elevation in HO-1 expression and decreases in the expression of p-p38, p-p65, and COX-2.

ASD contains not only sand grains but also various substances such as heavy metals, nanoscale particles, and organic chemicals generated by rapid industrial development [2]. Therefore, ASD exposure causes various harmful conditions in many organs and worsens underlying diseases [29]. In particular, exposing the respiratory tract to ASD induces inflammatory responses and aggravates underlying respiratory diseases, such as asthma and chronic obstructive pulmonary disease [23,30]. In acute lung inflammation, neutrophils migrate from the blood vessels into the lung parenchyma, where they directly engulf pathogens and stimulate the production of pro-inflammatory cytokines by phagocytic cells [31]. Pro-inflammatory cytokines, including IL-1, IL-6, IL-8, and TNF, mediate the inflammatory response [32]. Exposure to ASD has been shown to increase the production of pro-inflammatory cytokines in both mouse and human respiratory cell lines [24,28,33], and similar effects have been observed with exposure to silica nanoparticles, which constitute the majority of ASD [20]. In this study, ASD exposure induced pulmonary inflammation, with an elevation in inflammatory cell counts and inflammatory cytokines. However, silibinin treatment alleviated the pathophysiological alterations induced by ASD exposure. In previous studies, silibinin suppressed the progression of inflammatory responses in various inflammation-related conditions under experimental conditions, such as periodontitis, acute liver injury, and dermatitis [34,35,36]. Additionally, silibinin has also been shown to alleviate inflammation in various respiratory inflammatory conditions [17,18,19,20,37]. Therefore, our results indicated that silibinin effectively inhibits the inflammatory responses of pulmonary tissue caused by ASD exposure.

The inflammatory response is a defense mechanism used to protect the body from various stimuli, reducing harmful effects on the body. However, an excessive inflammatory response induces the development of various diseases and aggravates underlying diseases related to damaged lesions. During the progression of inflammation, various signaling pathways are involved [38]. Specifically, the NF-κB signaling pathway acts as a pivotal player [39]. NF-κB is phosphorylated by many stimuli and translocated into the nucleus, which eventually produces the proteins related to the inflammatory process, such as cytokines and chemokines [40]. A previous study showed the activation of NF-κB in response to ASD exposure [41]. Therefore, the control of p-p65 is an important therapeutic target to reduce inflammatory response. In this study, ASD induced the aforementioned phosphorylation and translocation into the nucleus, with an elevation in the expression of p-p38 and COX-2, which were diminished by silibinin treatment in both in vivo and in vitro experiments. These results indicated that silibinin inhibited the expression of p-p65 caused by ASD exposure. These were consistent with previous reports [9,19,42,43]. Silibinin exhibited protective or therapeutic effects on inflammatory conditions caused by lipopolysaccharide via the suppression of p-p65 expression [42]. Therefore, our results suggested that the therapeutic effect of silibinin against pulmonary inflammation caused by ASD exposure is related to the reduction it causes in p-p65 expression.

Due to the various irritating components of ASD, it induces oxidative stress on damaged lesions via the production of reactive oxygens species (ROS) [2]. The excessive generation of ROS induces an imbalance between oxidant and antioxidant systems, which leads to harmful effects on cellular molecules including DNA, lipids, and proteins, resulting in the alteration of normal cellular structure and functions [44]. Exposure to ASD has been shown to increase ROS production in human lung fibroblast cells [41]. Bio-organisms have various antioxidant defense mechanisms to prevent oxidative stress, among which HO-1 is an important signaling pathway for reducing oxidative stress [45]. HO-1 catalyzes the initial and rate-limiting steps in the degradation of heme to bilirubin, which has potent antioxidant activity [24]. Therefore, the elevation of HO-1 in pathological conditions induces a therapeutic effect by reducing oxidative stress [46]. In this study, silibinin markedly decreased the expression of oxidative stress marker 8-OHdG, and elevated HO-1 expression in pulmonary tissue. These results are supported by those of a previous study [47]. Silibinin reduced cognitive impairment in models of Alzheimer’s disease, and this is related to its antioxidant properties, which increase HO-1 expression [47]. Therefore, our results indicated that silibinin suppressed oxidative stress caused by ASD exposure via the enhancement of HO-1 expression.

## 5. Conclusions

Overall, silibinin treatment reduced the expression of p-p65, NF-κB, COX-2, and p-p38 in response to ASD exposure, while simultaneously increasing HO-1 expression both in vitro and in vivo, indicating that silibinin effectively suppresses pulmonary inflammation and oxidative stress caused by ASD exposure. Our results suggest that silibinin may therefore be a potential therapeutic material for treating pulmonary inflammation caused by ASD exposure.

## Data Availability

Data are contained within the article.
